# Evaluating the Control of Money Laundering and Its Underlying Offences: the Search for Meaningful Data

**DOI:** 10.1007/s11417-020-09319-y

**Published:** 2020-05-20

**Authors:** Michael Levi

**Affiliations:** School of Social Sciences, Cardiff University, Glamorgan Building, King Edward VII Avenue, Cardiff, Wales CF10 3WT UK

**Keywords:** Anti-money laundering, Money laundering, Organized crime, Financial crime, Risk assessment, Effectiveness measurement

## Abstract

This article examines the political and criminological history of anti-money controls, including the involvement of Asian countries and the varied motivations and interests engaged. It goes on to review where empirical evidence has been used and can be used in fighting the financial components of underlying crimes, examining the denotation of the problem(s) we are supposed to be fighting—money laundering as an evil in itself and/or the underlying crimes which give rise to it; estimates of the size and harmfulness of the problems and identifies any improvements/emerging problems in the assessment of money laundering. It concludes with some reflections on assessing policy effectiveness and the need for greater engagement by criminologists and other social scientists in this process.

And what rough beast, its hour come round at last, slouches towards Bethlehem to be born

William Butler Yeats (1920) *The Second Coming*

## Introduction: the Struggles of Anti-Money Laundering to Gain Traction

Special sorts of conditions must exist for the creation of the special sort of criminal that he typified. I have tried to define those conditions–but unsuccessfully. All I do know is that while might is right, while chaos and anarchy masquerade as order and enlightenment, those conditions will obtain.

Eric Ambler (1937) *A Coffin for Dimitrios*

In 1991, *Crime, Law and Social Change* published its longest ever single article, at just over 40,000 words: it was a review of the shaping of the anti-money laundering (AML) movement, at that time little more than a twinkle in Panopticon’s eye. It began by observing (Levi 1991: 217):

The principal points of contact between police and bankers are in relation to (1) frauds (and other crimes such as robbery) against banks themselves, in which banks lose money, and (2) crimes or suspected crimes - involving dishonesty, import/export prohibitions (e.g. as I write [in 1990], arms to Iraq), narcotics, or terrorism - whose perpetrators and victims have individual or corporate bank accounts, even though in such cases, the banks themselves may not lose any money and may even make profits from handling the accounts. There are also further areas of state interest in banking transactions, such as the activities of arms dealers who may not be committing any offences in the U.K. or elsewhere, but who are of concern to the security services.

The movement in the direction of encouraging - and in an increasing range of cases, requiring – ‘active citizenship’ on the part of banks has as its objectives to prevent criminals from benefiting financially (a) from the offences for which they have been convicted, and (b) to the extent that monetary gain is the criminals’ primary goal or is a crucial means to their other (e.g. political) goals, to deter or prevent them and others from committing crimes for gain in the future. These objectives are being pursued not only in Britain but also in the international arena.

Why should bankers co-operate with the police? Motives for co-operation can be placed on a continuum between positive - as when natural and/or corporate persons all hope to get something they want out of the relationship, even if the benefits are unequal - and negative - if the relationship arises solely out of fear or threat that something bad will happen to either or to both parties. Many non-banking professionals such as lawyers and accountants responded to my announcement of this research topic by asking, rhetorically, ‘what relationships?’ But in practice, police-bank relationships are characterized by a combination of both positive and negative elements.

Since that was written 30 years ago, the world has witnessed an extraordinary growth in efforts to control crime for economic and political gain (and, especially since ‘9/11’, for terrorism) via measures to identify, freeze and confiscate the proceeds of crime nationally and transnationally. Especially in Europe but in different ways throughout the globe, first bankers and then accountants, lawyers, notaries, real estate agents and even car dealers have been involuntarily co-opted into becoming unpaid agents of the state: identifying customers (individual and corporate) and transforming conventional legal constructs such as customer confidentiality, reporting both their suspicions (if they have them) and large cash transactions to central bodies. There is pressure from international bodies and national governments to get an increasing number of reports from a bigger range of commercial and professional actors. But what happens afterwards to these reports is far less transparent and—especially in those jurisdictions where there are the most reports—is frequently unproductive in visible outcomes such as prosecutions, asset recoveries and the prevention of serious crimes. The cost of AML implementation in the public and (mostly) in the private sector dwarfs the quantity of crime proceeds recovered.

One useful way of conceptualising the issue is as a global exercise in crime risk management which seeks to drag in as a conscript army those governments and those parts of ‘the’ private sector that seem unwilling to volunteer for transnational social responsibility. But what sorts of risks are they managing (and do they *believe* they are managing), and how politically coherent, serious and well-considered has the attempt been? The aims of nations (especially the original key movers—the USA, UK and France, with Switzerland and Australia also playing an important role) can change over time, but it is easy to over-estimate their coherence and stability over time. Interviews and documentation collected by the Levi (2000–2003) suggest that there were national issues that gave rise to particular foci of control interest—sometimes, like ‘drug abuse’, independently in different countries—before any coordinated international activity was even contemplated. Thus, prior to the UN Vienna Drugs Convention of 1988, the Swiss Banking Commission sought to regulate capital flight, and (post-Watergate) the Americans criminalised the bribery of public officials overseas, while the Americans and British (but almost no other countries at the time) criminalised drugs money laundering. These actions were prompted by concrete ambitions and sometimes by international scandals such as bad publicity for Switzerland as a ‘bank secrecy haven for kleptocrats’ and for the UK’s Crown Dependencies and Overseas Territories (and, to a less publicised extent, the Dutch Antilles ex-colonies) as ‘corporate secrecy havens’.

As regards Asian involvement in AML, as early as 1990, Japan joined the Financial Action Task Force (FATF), which was created in 1989, followed by Hong Kong in 1991 and Singapore in 1992. They were not major players in policy formation, but were welcomed as major financial hubs, and partly also to legitimate the idea of FATF as a global body: it became a permanent body only 30 years later, in 2019. The Russian Federation joined FATF in 2003, China in 2007, the Republic of (South) Korea in 2009, India in 2010 and Malaysia in 2016, reflecting both the size of their economies and their political importance, as well as their readiness (within limits) to buy into the global AML protocols. Those are the only Asian countries in FATF, though Indonesia is also currently an observer, normally a prelude to full membership. This has been a conscious geographical expansion by FATF, and the rise in Asian membership reflects the shift to the Far East in economic growth and power. Thus, while much focus has been on Asia as a production hub in the global supply chain for a vast array of goods, it has also risen financially. Looking at it from a financialisation rather than industrial perspective, in the well-established Global Financial Centres Index (2020), Asian countries now occupy the top seven places within the Asia Pacific region, and only New York and (pre-Brexit) London rank higher in the world (see Table 1).^1^ Though Hong Kong’s standing has fallen since the previous year, five Asian centres are now within ten points of London in the calculations. This table and others in the report may not be an indicator of centres’ respective roles in laundering the proceeds of crimes committed domestically or internationally, but it does reflect changes in the financial and political orientation of the international AML bodies and the need to ensure broader compliance in Asia.

**Table 1:** Asian city rankings in Global Financial Centres Index, 2019

Centre	GFCI 27 rank	GFCI 27 rating	Rank (+/−)	Rating (+/−)	Region
Tokyo	3	741	3	− 16	Asia/Pacific
Shanghai	4	740	1	− 21	Asia/Pacific
Singapore	5	738	− 1	− 24	Asia/Pacific
Hong Kong	6	737	− 3	− 34	Asia/Pacific
Beijing	7	734	0	− 14	Asia/Pacific
Shenzhen	11	722	− 2	− 17	Asia/Pacific
Guangzhou	19	714	4	3	Asia/Pacific
Sydney	20	713	− 10	− 25	Asia/Pacific
Melbourne	21	712	− 2	− 8	Asia/Pacific
Wellington	31	697	0	4	Asia/Pacific
Seoul	33	694	3	17	Asia/Pacific
Kuala Lumpur	44	677	1	17	Asia/Pacific
Mumbai	45	672	27	63	Asia/Pacific
Busan	51	664	− 8	2	Asia/Pacific
Bangkok	58	657	− 8	8	Asia/Pacific
Osaka	59	656	− 32	− 49	Asia/Pacific
New Delhi	69	646	21	58	Asia/Pacific
Chengdu	74	641	− 1	33	Asia/Pacific
Taipei	75	640	− 41	− 47	Asia/Pacific
GIFT City, Gujarat	82	633	− 16	9	Asia/Pacific

Even before the ‘9/11’ attacks and the corporate scandals of that decade, this author’s interviews and participant observation in international meetings since the 1980s suggest that the expressed alarm of the authorities was motivated by far more than institutional and personal self-interest and by ideological ‘control freakery’ that there is a genuine fear of loss of control over financial flows and of illegal drugs, which brings together a variety of themes and a variety of political positions including

1. financial regulators concerned about unmonitored ‘off the books’ (though often legal) transactions conducted by vast commodities hedging funds held in less transparent offshore finance centres;
2. law enforcement agencies and politicians bothered about ‘transnational organised crime’ and its ability to launder billions of what a senior former British civil servant termed in an interview ‘mutant capital’;
3. corporations (especially American-headquartered ones prohibited from paying bribes to foreign public officials by the Foreign Corrupt Practices Act 1977) campaigning for a legal ‘level playing field’ so that they can avoid losing tenders for contracts to bribe-payers;
4. overseas aid agencies troubled by the ‘export’ (aka theft) of funds by Third World potentates (Politically Exposed Persons, in AML terminology) into covert individual and corporate accounts and luxury property held in offshore finance centres (e.g. Sharman 2017)^2^ and
5. corporations, intelligence and law enforcement agencies, and politicians concerned about terrorist finance both from proceeds of crime *and* from legal-source income.

A research visit by this author just prior to the ‘9/11’ attacks discerned almost no interest in money laundering within the World Bank, and relatively little positive active interest in the IMF either, despite the strong general focus on anti-corruption and governance issues in the World Bank Literature. So, it would be a mistake to ‘back-read’ the current confluence of interest in money laundering as a natural reflection of those specialised institutional interests: it took a determined effort (a) to get the anti-corruption movement to see the relevance of money laundering and (b) to persuade the IMF of money laundering’s (still disputed) macro-economic relevance to its formal mandate.

More recently, President Xi’s strong interest in promoting the anti-corruption drive against both ‘flies’ and ‘tigers’ to reduce the illegitimacy risks to the Communist Party from actual and perceived rampant corruption has placed this issue centre stage, and China holds the Presidency of FATF 2019–2020, with the proclaimed objectives of

- Conducting a strategic review
- Mitigating the risks and exploiting the opportunities of new technologies
- Promoting and enabling more effective supervision by national authorities
- Other objectives:
  - Strengthen coordination and capacity for training on the FATF standards throughout the FATF Global Network
  - Action on illegal trafficking in wildlife
  - Develop and issue best practices on beneficial ownership^3^

(Though some nations including China, Russia and the USA might have difficulty convincing sceptics that their targets for investigation and prosecution were impartially selected for their culpability and harmfulness, and were not influenced by political and/or personal considerations.)

In the more general setting of the AML movement, we can see the interplay of crime control, personal and institutional dynamics. Early Italian interest in the use of proceeds of bank robberies to fund the activities of the *Brigate Rossi* in the late 1970s drove *local* requirements on Italian banks to report large cash deposits, and the realisation that this would make sense across borders and be applied to the Council of Europe generally. However at that time, there was insufficient political support for a binding convention, and the more modest recommendation was made which in turn fed into the justification of the first general measure against money laundering and asset confiscation in Europe—the Strasbourg Convention of 1990—which went beyond the drugs-only focus of the 1988 UN Vienna Convention because it was already apparent to the Council of Europe Secretariat and to countries such as the UK that the drugs-only approach (which was so necessary to get agreement in the consensus atmosphere of the UN) was a theory failure: not only did it not go far enough for a serious measure of major crime control, but also it left too much room for ‘launderers’ and financial intermediaries such as banks to claim that they did not know that the funds were proceeds of drug trafficking.

The key Council of Europe Secretariat member Hans Nilsson then joined the Secretariat of the Council of the European Union (at that time, a tiny activity long before the recent development of the Justice, Home Affairs and Migration functions) and brought with him a batch of ambitions to develop not just laundering and proceeds of crime but the entire range of mutual legal assistance provisions, plus the monitoring of compliance that was a key component of the FATF. At that time, however, the European Commission had no competence in criminal matters, so controls of money laundering had to be smuggled in under the (still largely unexamined) guise that variations in Member State provisions against laundering were a threat to the integrity of and free flow of funds within the internal market. This was a fiction that most Member States were happy to go along with (though many did not see it as a fiction), since the Treasury officials of the larger States had already been led by the FATF into thinking that AML was part of their role, and it was difficult politically to be seen to support money laundering, particularly when the measures of control were not obviously severe. When this author was rapporteur at a UN conference in 1994 at Courmayeur, it was clear that China and Russia wanted to restrict AML to proceeds of drug trafficking, but this reflected their crime and political concerns at the time, and AML has now extended universally to all crimes, including overseas tax evasion and financing ‘terrorism’—a much disputed term around the world because of its value in suppressing and de-legitimating minority struggles.

Also important in the evolution of AML activities were more modest elements, well below the threshold of grand social movements or international diplomacy. Prior to the Treaty of Amsterdam, the European Commission had no formal policy development and initiation role in crime control issues. However, the encouragement by funding of mutual assistance programmes under the principle of information exchange, combined with sharing of experiences by members of FATF (a permanent body from 2019) and Egmont—a global semi-official organisation of Financial Intelligence Units whose secretariat currently is in Toronto, which has grown from 12 members in 1995 (when it began) to 101 in July 2005 and 164 in 2020—generates a level of mutual knowledge at a personal and perhaps legal system level that encourages interpenetration, though this is easier to do within common law and within civil code systems than between them. The increased ‘traffic’ between countries forces them at an operational level to seek solutions to their interface problems (though these do not always succeed or are they always intended to). Thus, Egmont was generated originally by the need of the American and Belgian administrative type financial intelligence units (FIUs) to both obtain information from and transmit information to police-type FIUs that existed elsewhere in the world (Today, this issue still causes difficulties, for example in Germany, at a domestic as well as international level.). So it would be a mistake to see these policies and practice mobilities/transfers as coming either from above or from below: rather they come from both, though given the financial cost of many exchanges in the money trail arena, some positive support from senior officials is often needed before the exchanges are allowed to develop. In this sense, international policing is different from local partnership policing, where few marginal costs are involved: mid-week plane fares, hotels and time off work are considerably more expensive and generally cannot be found from routine or training budgets. Note, however, that except for regular formal meetings, such extra funds tend to be available only for activities related to ‘organised crime’ or ‘terrorism financing’ as commonly understood. So, if these issues had not struck a political chord, some of these developments might have been strangled for lack of finance.

In more recent years, the European Commission has been involved in supporting action against organised crime and payment fraud, and to sponsor private-public partnerships in organised crime and terrorism prevention, as well as manifold legal measures. The other key role of the EU has been in relation to the *acquis communautaire*, which has enabled them to engage in pressurised policy transfer of AML, anti-corruption and mutual legal assistance legislation and programmes to candidate countries. This has involved self-assessment questionnaires, mutual evaluations (also making use of the MONEYVAL and GRECO evaluations within the Council of Europe framework) and some technical assistance programmes. No other region has been able to exert analogous pressure, based on shared values (currently under strain from Hungary and Poland), and aside from the lack of institutional leverage, it is doubtful if sufficient mutuality and trust exists within Asia. The USA has long exerted its own pressures unilaterally and multi-laterally, and this is not the place to review its efforts.

The history of money laundering and its control has until recently been subjected to very little *intellectual* debate, nor does it have any major theoretical drivers beyond a common sense appreciation that without ‘fences’ (receivers of stolen goods) there would be few thieves, and without the ability to hide, re-invest and safely use proceeds of crime, there would be less crime (or perhaps the same amount of crime committed by a larger number of criminals, since storing large amounts of criminal wealth would be too difficult) (Levi and Reuter, 2006; Nance 2018). In this sense, it has never aspired to the procedural rigour recommended by the OECD (2020) on Regulatory Impact Analysis. Most of the political drivers began in the USA, with efforts by the (Reagan) US Presidential Commission on Organized Crime (1986) to get investigators to ‘follow the money’, but there was almost no input from academic criminologists or economists into bolstering the anti-money laundering movement, whatever modest input some individuals may have had on shaping the struggle against organised crime. Nor did academic critics and/or empirical research have any noticeable effect on slowing the AML movement down, despite the private reservations of some law enforcement officials and professionals (Naylor 1999; van Duyne et al. 2019), though even with the EU, there are differences of emphasis and structure related to national cultures and principles of law (Vogel and Maillart 2020).

These judgments are not merely rhetorical. The period 2008–2019 led to regulatory fines on financial institutions totalling US $36 billion. Two countries—Iran and North Korea—are ‘blacklisted’ by FATF, meaning that no bank or regulated person in the world is allowed to deal with them without committing money laundering and/or sanction offences which could lead to their being imprisoned or closed down. There are a wider group of countries that are under periodically reviewed ‘grey-listing’ as ‘Jurisdictions with strategic deficiencies’ (http://www.fatf-gafi.org/publications/high-risk-and-other-monitored-jurisdictions/documents/increased-monitoring-february-2020.html). In Asia, these include Cambodia, Mongolia, Myanmar and Pakistan among the 18 countries currently (May 2020) ‘under monitoring’, none of which could properly be described as systemically important economically. Being thus listed can have significant effects in making it harder and more expensive to find banks willing to process national transactions and to open accounts with international banks (‘correspondent banking’): ‘de-risking’ in response to classification as a ‘money laundering risk’ is acknowledged by the IMF, World Bank and Global South countries to be a problem of concern.

## The Use of Evidence in Fighting Crimes for Gain

The appropriateness of a strategy depends on where we want to get to, and on how (if at all) we can get there from where we are now. Beyond activities and hopes, it has never been clear at any stage of its development where the loose-coupled AML ‘system’ has wanted to get to (beyond ‘more of what we have been doing’), or what empirics have been either desirable or feasible to help it reach ‘its’ goals. This does not appear to have inhibited its appeal to policy makers, enforcement agencies, or—arguably—the various publics, though there is little evidence about what if anything the public understand and believe about AML. Indeed, its very lack of clarity of objectives may be part of its sustained appeal. AML gains support from many interests on the political left and right; and there has never been any serious attempt to examine what it would cost—and to whom—to satisfy or even to satisfice these varied constituencies.

The data that are relevant to system goals are ill-expressed, and goal under-conceptualisation has not assisted. Data have several components, including laundering the proceeds of domestic crimes and crimes committed elsewhere, and evidence that links the process of AML to changes in these predicate crimes and/or their organisation. These have different patterns, and knowledge about where the money passes through and where it ends up are very patchy (Levi and Soudijn 2020).

In addition to the rejection of potential customers and the ‘de-risking’ (i.e. closure of accounts) of current individual, business and charity customers (which themselves are seldom linked explicitly to crime reduction), a process map of Counter Criminal Finance measures (beyond front end prevention such as de-risking) might look like this:

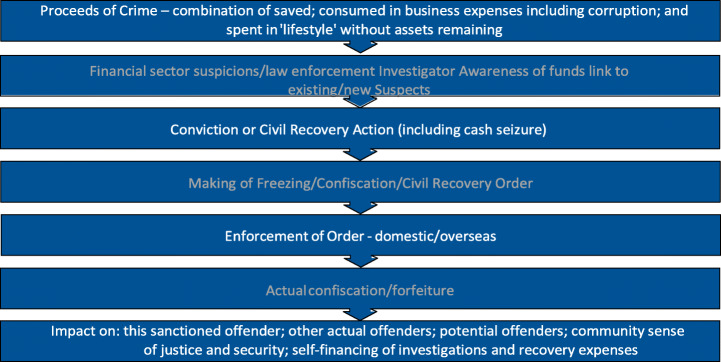



Some components of the above are matters upon which data can be collected retrospectively or, to be more realistic about the costs and effort of data-gathering, prospectively, though data reviews would require a longitudinal flow rather than an annualised method of accounting, since the process of proceeds of crime identification and recovery may often take place over longer than a pre-defined or an elapsed time year: a fact that has received comparatively little attention to date. Life-course criminology has paid attention to some features of organised and white-collar crime careers, but not yet in the context of the impact of AML measures. As we shall see, it is far easier to critique the data that exist than it is to work out and to create the empirics that illuminate effectiveness, and one question should be whether (as in drug policy) governments individually or collectively really *want* to evaluate effectiveness and to adjust their policies in the light of it. ‘Crime control’ is often a matter of expressing values or signalling virtue rather than reflecting evidence from science.^4^ Signal crimes (Innes 2014) are more salient than crime data to most politicians and priority setters.

First, however, we need to look at what is meant by financial crime and the harms that it brings. These are treated quite casually both conceptually and in practice in the international money laundering literature, though measuring illicit financial flows has become a popular theme among economists in recent years.

## Definitions of Financial Crime and the Beginnings of Effectiveness Assessment

‘Financial crime’ is normally not a legal but rather an administratively defined category. Its use has been growing in the Global North, though in continental Europe and parts of Africa, ‘economic crime’ is more commonly used and overlaps extensively. Almost two decades ago, an IMF (2001: 3) paper defined it very broadly, co-extensively with what would now be money laundering:

Financial crime, which is a subset of financial abuse, can refer to any non-violent crime that generally results in a financial loss, including financial fraud. It also includes a range of illegal activities such as money laundering and tax evasion. Money laundering refers to activities involving the processing of criminal proceeds to disguise their association with criminal activities.

So too did the UNODC (2005: 1) at the 11th UN Crime Congress:

‘[E]conomic and financial crime’ refers broadly to any non-violent crime that results in a financial loss.…The category of ‘economic crime’ is hard to define and its exact conceptualization remains a challenge.

Note that taken literally, this would include all thefts and all burglaries of empty premises. There is no evidence that any of the global figures generated and recycled endlessly (e.g. UNODC 2011) as ‘facts by repetition’ are used operationally as a baseline for assessing the effectiveness of AML or of any other policy: if it was, as with the various forms of ‘wars on drugs’, the policy framework might have to be abandoned or seriously modified. Even in those countries where evidence-based policing is not a mantra, however, *some* performance data are called for, though countries struggle to utilise sensibly their analytical value as proxies for effectiveness. In principle, jurisdictions opt into FATF-Style Regional Bodies (FSRB) membership and into the ‘mutual evaluation’ process by other countries in their region (or world in the case of FATF). On a rota, nearly all jurisdictions in the world have to be evaluated at least every decade by the FATF or FSRB (the Asia Pacific Group, in the case of most Asian countries). The ‘mutual evaluations’ ask countries to show evidence of performance in AML by, e.g. the number of Suspicious Activity Reports (SARs), Suspicious Transaction Reports (STRs) or routine financial transfer reports made to the national Financial Intelligence Unit, criminal laundering prosecutions (including standalone ones), asset freezing and confiscation, etc.: but preventative activities and their impacts are assumed rather than measured.

The easiest way to please the evaluators is to prosecute more self-laundering cases (independent of drug trafficking or fraud prosecutions) and thus increase *recorded* ‘money laundering’ and ‘financial crimes’, accompanied by a narrative of how this reflects their greater efforts against laundering. Halliday et al. (2014) were rather critical of the earlier Third Round FATF evaluation process, likening it at times to the Russian Tsarist Potemkin Villages, in which an appearance of compliance and formal structures were often sufficient to impress. It remains an open question to what extent this has changed and will change further in the formally more outcome-focused Fourth Round using the significantly revised FATF (2013) Methodology which aimed to offer a greater assessment of real world effectiveness. What is clear is that the majority of countries have begun already to conduct the National Risk Assessments they are encouraged to carry out based around as a prelude to this evaluation process, though consultants with experience of using (or in some cases, gaming) the system to gain better marks tend to be brought in—mainly after the NRAs—to help the jurisdiction prepare for the mutual evaluation. Since there is little analytical understanding of what actually might affect the impact of AML on crimes, consultancy expertise is more likely to be how to please evaluators.

The criminalisation of transnational (and sometimes national) bribery affects banks directly via risks (if they ‘fail’ to report any suspicions they may have) of money laundering charges (a) against their own individual Money Laundering Reporting Officers and perhaps (b) against the banks themselves. It also affects banks indirectly because of the risks (however remote in practice) that companies to whom they have lent money might be damaged by severe penalties should *they* be convicted of corruption. The implications of these developments are important.

As used in different countries, ‘financial crime’ and ‘economic crime’ comprise crimes with different categories and levels of harm, committed by and impacting upon highly diverse sectors of the population. Their sub-components are investigated (and investigatable) by very different policing and regulatory methodologies both before and after ‘crime’ commission. As a legal category, ‘laundering’ does not enable us to distinguish between licenced professionals like lawyers who launder, professional (i.e. regular) knowing money launderers, people who launder money from their own crimes (like burglars putting money into their own bank accounts in their own names) and banks who intentionally or recklessly ignore their obligations to report suspected money laundering, or who turn a wilfully blind eye to ‘smurfing’ by customers who deliberately place money in their accounts that fall below the reporting threshold (e.g. $10,000) (Levi and Soudijn 2020). Note that the term ‘banks’ here is an anthropomorphic construct of entities of varied complexity and transnational integration that range from relatively small local banks to HSBC (which in 2018 had 238,000 employees in 65 countries and territories) and Citigroup (which had 214,000 employees in 160 countries). Banks generally counter that any laundering is the result of rotten apple individual or small group behaviour rather than institutionally supported misconduct, and without smoking gun emails/recorded conversations, or verified whistle-blower accounts, or some ‘market testing’ mystery shopping exercise as conducted by Findley et al. (2014), it is difficult to test this or validly falsify such claims. What is certain, however, is that violations by institutions great and small continue, despite occasional regulatory and rare criminal institutional sanctions. Some more subtle thinking is needed on metrics of reoffending also, for example given their size, should we treat a violation at HSBC as being ‘the same’ as one at a much smaller institution. After all, we would expect risks to be higher among those who do much more activity.

### Searching for Appropriate Crime Data: the Role of the National Risk Assessment

In this era of globalisation, problems also arise for crime statisticians. One could question how meaningful it is to use the nation state as the unit of harm and threat, but this has not been posed explicitly in the AML debate. Jurisdictions are encouraged to conduct National Risk Assessments, presumably with their own country’s risks—i.e. threats and vulnerabilities—as the central organising concept, though any proper analysis of money laundering risks should contain analysis of the risks posed to other countries as well as one’s own. For example, an analysis of the threat posed by money laundering in the casinos in Macau (or, for that matter, in the casinos of Australia or in Canada’s British Columbia—see German 2018, 2019) cannot properly ignore the threat *to* China from corruption, embezzlement and other forms of crime occurring *in* China, even if there was little risk of this laundering increasing domestic crimes in Macau, Australia and Canada. Besides, it seems likely that much gambling in Asia and elsewhere is carried out because criminals like gambling rather than primarily to launder money, though tighter controls on identifying gamblers might frighten them off gambling in regulated places: what impact such controls would have on levels of criminality is an open question, especially if they could easily engage in illegal underground gambling which might increase their liability to blackmail and mixing between public officials, ‘pure’ financial criminals and ‘organised criminals’.

There is no requirement in the FATF standards to undertake a *written* assessment of risks. The standard (Recommendation 1 in particular) requires only that countries ‘identify, assess and understand’ risks, not that they actually prepare a written document. Thus, a country can have a variety of documents or instruments that collectively may satisfy the requirements of the FATF standards but which, even when taken together, do not constitute a serious National Risk Assessment. Many Global South countries in Asia and elsewhere do not publish their NRAs, and those that do, publish only sanitised versions: it is moot whether the classified versions are significantly more informative, not least because though they may contain more detailed cases and names, it is uncertain what data they could collect easily would illuminate the risks and weaknesses better. Thus, publicly available data on crimes and how they are used to address money laundering risks are extremely patchy, even in FATF countries, let alone in Asian and other countries where criminological sophistication and an appetite for openness may be low. As the FATF Mutual Evaluation Report for China (2019, para 10) put it:

Overall authorities in China demonstrated a strong understanding of the contents of the NRA which was finalized just prior to the on-site visit. However, given the focus of the NRA and the activity of LEAs, on predicate offences and the lack of attention to how the proceeds of crime are actually laundered, beyond those directly implicated in the crime, China’s overall understanding of ML risks, while achieved to a large extent, is hampered by such a focus. The assessment of risks of legal entities focuses on existing control measures. The TF [terror finance] assessment contained within the NRA is based mainly on qualitative analysis. The analysis collates information from departments involved in countering terrorism, primarily MSS, MPS and the PBC, identifying sources and channels of terrorist financing, and identifying the TF threats faced by China.

Indeed, it is often difficult to find a strong awareness of how money laundering is accomplished, beyond general categorisations (termed ‘typologies’ in AML speak) or individual cases often cited to show what kind of work is being done (Levi and Soudijn 2020). Focusing on FATF members in Asia, China’s NRA has not been published, though Hong Kong’s 2018 NRA has. Singapore’s NRA was conducted in 2013 and no subsequent NRA has been published: it asserts that despite high inherent risks as a destination for international capital, “Singapore has in place a robust AML/CFT [Combating the Financing of Terrorism] regime, grounded in tough regulations, rigorous supervision and effective enforcement that has helped mitigate these risks. There are a few areas where controls need to be strengthened and efforts are underway to address these areas” (Singapore, 2013: 5). Malaysia’s NRAs have been published, but even the most recent one in 2018 contains no mention of the 1MDB scandal, one of the largest alleged embezzlements of public funds in history which has already brought down senior executives at Goldman Sachs bank and where in 2019–2020, the former Prime Minister Najib Razak has been on trial.^5^ Japan’s 2014 and 2018 NRAs are published, though there is little hint in them of what methodologies have been used to underlie these, a gap that is common even in G7 NRAs (Ferwerda and Reuter 2019). There are also EU supranational risk assessments in 2017 and 2019, though none yet for any other regional bloc, though the Asia-Pacific Group recently published one for non-profit organisations and terrorism finance risks, and has also done so for wildlife crimes.^6^ Many countries use a NRA ‘Methodology’ sold by the World Bank and some by the IMF, while others still devise their own, but very few give details, and all face the problem that if they are honest and self-critical, they may receive a lower grade than others who are less honest or insightful.

### Rethinking Risks and Measures in National Statistics

Normally, when we think of risk, we think of harms that can be plausibly or potentially done to a natural person or an organisation/legal person (and perhaps to a sector). This has commonly been extended to ‘reputational risk’ with its implications for individuals and businesses’ future capabilities and profits. We normally conceive of victim-offender or consensual crime (e.g. illegal drugs and sex) relationships—whether violent or for financial gain—as occurring *within* countries. However, many significant financial crimes (not *just* the often problematic and over-homogenised category ‘cybercrimes’—see Levi 2017, Kemp et al. 2020) occur between people in *different* legal jurisdictions, at least at some stages in what this author would term the criminal supply chain, from production/planning to money laundering. An improvement in laundering statistics therefore needs more breakdown of sub-types to make sense of these acts, which also are problematic to capture in *national* crime and justice statistics.

Arguably, if we followed Wilkins (1965), who considered that it might be fruitful to conceive of (recorded) ‘crime’ as more like complaints about which the public thought that ‘something should be done’,^7^ then there might be no money laundering offences at all. Civil Society Non-Governmental Organisation reports, e.g. from Transparency International (TI) or Global Witness, might be treated as equivalent to such bystander complaints, though only in the context of bribery and corruption, which constitute a tiny proportion by volume of SARs and a small share of the anti-crime goals of AML.

One might take SARs or STRs as a comparable indicator to the Wilkins model, since they are alerts to *possible* malefaction: but it would be reasonable to conclude that rather than reflecting reporters’ own normative beliefs that action *ought to* follow, SARs reflect regulated persons’ blind obedience to rules and/or fluctuating terror at being punished for *not* seeing that transactions are acts about which ‘something should be done’ by regulators or criminal courts exercising hindsight.^8^ But, it is worth noting that recording racially motivated offending/hate crime also involves a subjective victim or bystander judgement about motivation rather than an objective fact.^9^ However to my knowledge, no government or statistical office has yet taken seriously the task of creating criminal statistics for money laundering beyond prosecutions or convictions, which may be set out in justice statistics rather than in crime statistics. Indeed, a word search of UK Home Office and Ministry of Justice statistics (5 December 2019) yielded zero for money laundering.^10^

How in practice do we separate temporary resting places flows from ‘final destinations’ of money? Given the range of predicate crimes of poor estimatability and our ignorance about the savings ratios of offenders, even working out an order-of-magnitude figure with a large range raises the question of what is our motivation or perception of value for performing such an exercise (see Reuter 2013; van Duyne et al. 2019). Looking beyond the UK, which normally has the most adventurous and professional approach to crime statistics,^11^ Eurostat (2013) conducted some preliminary analysis of European data but stopped after its second edition. Its tables are almost all about process; its figures (p.4) are as follows: Declarations made in application to the EU Cash Control Regulation; Incorrect cash declarations or findings as a result of customs controls in the EU at external borders; Total number cases brought to prosecution originating from reporting by regulated bodies; Number of persons/legal entities convicted for ML offences. Improving data does not seem to have been an active priority for any national or international body. But the data are not even particularly useful for assessing efficiency, let alone effectiveness.

In its review of data on the national income implications of illegal activities in Europe, Eurostat (2018: 72) notes

For the purposes of this Handbook, money laundering is defined as a process transferring illegally obtained money or investment from illegal economic activities — those identified by the GNI Committee (i.e. production and trafficking of drugs, prostitution, and smuggling of alcohol and tobacco, based on transactions mutually agreed between consenting parties) and those discussed in Part II — through an outside party to conceal their true source, and make them (appear) legal. The difference between the value of the illegal money and the legalised money should be understood as the value of the provision of money laundering services (PMLS).

This does not explicitly distinguish between the price of laundering (i.e. what criminals are charged) and its value to offenders. What criminals are paying for is the usability of the proceeds of crime in licit markets and relative freedom from/anxiety about confiscation. There follows discussion of how money laundering data should be treated in national accounts, concluding with a somewhat optimistic reliance on evidence from an EC-funded project ECOLEF (Unger et al. 2013) which examined the attractiveness of EU countries to money laundering (or ‘risk’ in official language, perhaps ‘opportunity’ from a deviant perspective). The Eurostat Annexes use the 2009 data from the ECOLEF study, without considering any changes in attractiveness or scope in the intervening decade, relying on ‘the Walker gravity model’, a construct that has been more attractive to policy makers than to serious economic or criminological analysts (Reuter 2013). This risk of using outdated information is a common problem with episodic studies that are not built into the routines of data collection and analysis, or is there any awareness displayed that (as with some fraud) legal and social definition of activities as falling within or outside the definition of money laundering can be a highly contested decision, especially in large cases. However, despite the use of some dated material and a curious selection of references, it contains some interesting (to economists) material on how to integrate money laundering into national economic statistics and represents a decent starting point.

There have been more modest data-gathering exercises on particular dimensions of money laundering. One that held promise was the attempt to develop price discrepancy analysis for testing over and under-invoicing of physical goods (e.g. de Boyrie et al. 2004): the under- or overvaluation of virtual services and intellectual property in international transfers is harder to test. At a national income level, this can be important in countries which are entrepots for smuggling wholly illegal, counterfeit or tax-evaded products, from drugs to wildlife. National statisticians might have to try to consider issues of customs and high level bureaucratic corruption in trade in legal, counterfeit and wholly illegal commodities which might be career-threatening. Publicly, the price discrepancy model has not been verified or falsified as a model, and given the secrecy of customs/tax bodies, it is difficult to infer whether their proposals were impractical, or whether the political economy pressures or bureaucratic ones have inhibited take up. There has been little public or media clamour for evidence-led or even evidence-influenced policy in AML.

### The Harms of Money Laundering

Another contested area, often taken for granted in public rhetoric about money laundering, is the assessment of harm (see e.g. Ferwerda 2013). Conceptually, what is the marginal harm arising from the laundering of the proceeds of domestic crimes that is not also a harm of the crimes themselves? Bribery and/or blackmail of financial service staff, regulators and law enforcement may occur in any country. But as with prohibition of alcohol or drugs, some of those crimes may be an artefact of the criminalisation of laundering rather than the direct result of the laundering itself: in the ‘good old days’, such funds might simply have been accepted and used, so there was no need to bribe or pressurise staff, and bank regulators might have been interested in the loan book and internal theft risks but not in customer integrity except as fraud risks.(Some serious Asian banking problems have been the result of non-arm’s length ‘loans’ to politically well-connected businesspeople that then are defaulted or never pursued by the banks, but that is fraud or corruption, not just a laundering harm.). Al Capone and Meyer Lansky were laundering the proceeds of illegal gambling, extortion and alcohol trafficking before laundering was criminalised, presumably in order to make it more difficult for the authorities to link their proclaimed assets and themselves to crimes (and maybe also to evade taxes). Some fraudsters— in Asia and elsewhere—will seek havens where they and their assets will be protected and from which they can launch their scams, but this protection (including corruption but also lack of enforcement motivation) often accompanies crime commission as well as laundering.

Collectively, at a global level, the availability of secrecy havens may stimulate a range of crimes and it can lead to moral outrage at the unfairness of elite offenders ‘getting away’ with their corruption, tax or mainstream crimes. However, the laundering of crimes committed elsewhere might be regarded as socially harmful *in the laundering country* only if it undermines democratic controls and legitimacy and/or if it leads to grey-listing or black-listing if detected *and* reacted to by international bodies such as FATF, and by rating agencies which decide national creditworthiness and economic prospects. In other words, it is harmful if it leads to a ‘control wave’ rather than because it is part of a ‘crime wave’. *Past* ‘adverse media’ is a factor in which customers accepting and monitoring risk assessments by regulated persons, though except for those who decide to conduct ‘market intelligence’ as part of enhanced due diligence on natural or legal persons, *future* adverse media *predictions* are not commonly done to jurisdictions as a whole unless sanctioned by private sector rating agencies.^12^

### Alternative ‘Takes’ on ‘the Problem’

Money laundering is often (mis)taken as a coherent problem. Instead of treating one report as a total authoritative statement of the subject, we might take the approach of Kurosawa in his film about a rape in mediaeval Japan, *Rashomon*, take different cuts of the phenomenon in order to illuminate a multiplicity of perspectives. And then, to the extent analytically appropriate, put these together. Such an approach might also reflect the range of interests in AML as a ‘solution’ to the social problems they are highlighting.

One way of visualising money laundering statistics on a global basis is to take leaks such as the Panama Papers and what issues they illuminate (see also Obermayer and Obermaier 2017; Bullough 2018).^13^ It is not self-evident what inferences about money laundering could be drawn from this snapshot of incorporations data: licit tax advantages or convenience could dictate an unknown portion of them, and it is not known what proportion of the ‘offshore’ companies was used for illegal purposes (Most tax authorities’ actions remain secret, so the efforts of the International Consortium of Investigative Journalists to publicise their impact are struggling to enhance the public face of post-leak interventions globally or in Asia in particular. The suspicion remains that the revelations have been under-exploited by under-resourced and or politically muzzled tax and anti-corruption agencies.).

A different ‘take’ might involve a snapshot of the largest alleged embezzlers or kleptocrats, though the data would have to be annualised and adjusted for inflation and currency fluctuations, which does not appear to have been done here(see also Sharman 2017).^14^ Again, the evidential basis for these suspected or claimed kleptocratic totals is hard to test. Like many estimates coming from NGOs and even from IGOs like the IMF, World Bank and UNODC, their main impact is that they are big and therefore generate an action imperative and a claim on our moral consciences. These estimates are seldom if ever used as an effectiveness baseline, not least because they would be too demotivating for agencies and bad PR as a very small percentage of total crime proceeds/laundering. Instead, there is a preference for headline figures of amounts seized or confiscated, though even a million pounds would only just buy a median house in prime London (https://www.bloomberg.com/graphics/property-prices/london/). Three former Asian leaders are in this ‘top six’ alleged embezzlers in history and scattered their assets in multiple jurisdictions, including some close to home because if you wish to remain in power, you usually need to sweeten political constituencies and/or other elites (Levi 2012; Sharman 2017).

## Reflections on Effectiveness

Effectiveness is a term that is readily used in policy circles, for understandable reasons most of us want to believe and get others to believe that our actions are having a positive effect. However, it often entails a confusion between *activity*—legislating or arresting or seizing assets or ‘doing something’ about a problem—and *outcomes* such as less crime, less ‘organised’ crime, or less social harm. To a populist politician, the popularity of a display of effort against something may be sufficient as an outcome, though few are brave (or perhaps even aware) enough to declaim that explicitly as their *only* goal. But, there are few criminal justice policies whose impact on *crime* can be so easily and quickly judged. And the more complicated and broad the target offences are, the longer is likely to be the time taken for evaluation and for the linkages between actions and effects. Furthermore, whereas we might measure economic transfers (e.g. burglary, robbery, theft, some frauds) by crime/victim surveys of the general population or sub-populations, service crimes (illegal drugs, sex, gambling) are harder to measure accurately, though they can be measured by anonymous self-report general population surveys. Measuring the involvement of a range of actors in money laundering is harder still, since these may include not only ‘underground’ businesses but also mainly legitimate players whose *intentional* wrongdoing would be deeply contested even if there were prima facie evidence of their involvement.^15^ If one broadens the lens, one might look at Google, Facebook and other social media platforms who are paid by scammers and others to advertise and trick victims, or to engage potential money mules for laundering proceeds of any crimes (e.g. from Chinese underground banking via Chinese students internationally— NCA 2019). If paid from proceeds of crime, they are properly labelled as ‘professional enablers’ (see generally, Levi and Soudijn 2020), though this label does not imply that they have the level of intention or recklessness to be prosecutable for money laundering offences.

Whatever the rhetoric of organised crime, the logic has seldom been carried through of treating money laundering as a *system*, beyond individual investigations of ‘core nominals’, ‘Mr. Bigs’, Designated Drug Trafficking Organizations or individual targets. The closest to this systematic approach for finance is the US Geographic Targeting Orders.^16^ Whether the same metrics should be used for different forms of crime is an open question, and may need close attention to the routes through which illicit products pass and who needs to be paid where for their contributions to the particular crime patterns (which can change over time). Beyond media headlines, long prison sentences for individuals or the direct economic costs of large financial penalties and monitorships against financial intermediaries, the impacts of such approaches are unclear, except on employment and consultancy income in the AML sector, which benefit substantially. ‘Policing for profit’ asset confiscation obviously stimulates investigative activity, but the positive and negative effects of that on levels and organisation of different crimes are only partially understood.

The total elimination of serious and organised crime of corruption and/or money laundering is too severe, a test against which to judge policy effectiveness, and the collateral damage from high effectiveness would be likely to be intolerable (as it is for some other offending also). Even if the number of crimes remains the same, a reduction in the social threat posed by some combinations of criminals may be a positive move: though disrupting criminal monopolies/oligopolies can lead to more short-term violence, as friction between offenders is increased. But it is not currently viable to conclude that any type of crime has been substantially *reduced* let alone eliminated altogether by AML/CTF measures (Levi et al. 2018; Halliday et al. 2019). It is also important to be clear about what *component* has plausibly impacted on which crimes (as we noted earlier when teasing out the differences between the harms of predicate crimes and the harms of laundering). In short, despite the huge effort involved in combatting money laundering, extending ‘the state’ into the banking, car dealer, jeweller and (variably) the professional sectors like lawyers and accountants (FATF 2018; Middleton and Levi 2015; Levi and Soudijn 2020), working out what data we need to evaluate risks and intervention impacts remains a work in slow progress.

There are also some important issues of national (and personal/institutional) self-presentation: a good risk or threat assessment requires ruthless self-examination, yet being open about this can increase the risks of weaknesses actually being exploited (though whether rates of exploitation are real or merely functions of increased surveillance may be unknown). Self-criticism can lead to lower gradings from the FATF or FATF-style regional body, including being placed on the blacklist or grey list with serious economic consequences as well as closer monitoring. Given this, in addition to cultural issues such as national pride or shaming that are sometimes stated to be more important in Asia than elsewhere, it is hardly surprising that countries aim to present themselves in the best light (In theory, one can be ruthlessly self-critical in internal reviews, but put on a brave face externally, but this ignores the fact that without external pressures, it may be hard to get resources for action.).

There has been very little conceptual or empirical research funding for money laundering or AML/CFT compared with ‘mainstream’ areas of crime and crime control. In addition to thinking through how activities contribute to the reduction of particular forms of crime or threatening crime groups, much work is statistical and requires good datasets to play with and perhaps create. These do not exist in the public sphere, and academics have sought to do so laboriously at the national level, though within the UK as well as many Asian countries, even court and prosecution data are not routinely accessible or are not conveniently classified. Private commercial risk analysis bodies like Refinitiv and Lexis-Nexis do have datasets, at least on money laundering cases, which in principle feed into their risk assessments of countries and activities which influence decisions to take or reject clients, though the risk raters may not override the FATF judgments about the relative and absolute ‘riskiness’ of jurisdictions. Individual financial institutions and professions, and regulators, have much live data, but access is hard to obtain.

It seems unlikely that our data on a range of crimes and laundering mechanisms will ever be good enough to meet the rigours of full evidence-based policing standards (though if such methodological ‘Gold Standards’ are not appropriate or realistic for analysing some of the most important social problems in our societies, perhaps that suggests the standards need greater flexibility). However, one of their aims of this article is to get criminologists and other social scientists to engage with the conceptual as well as empirical issues involved in measuring crime problems and the connections between ‘anti-criminal finance’ interventions and criminal behaviours, as others have done in the analysis of drug markets (MacCoun and Reuter 2010; UNODC 2019). It may be time for us to devote more sceptical attention to claims about how money is laundered, to claims about the efficiency of AML processes and the likely effects on crime of efforts to combat laundering and the financing of terrorism. This should include attention to the possible counterproductive impacts (Grabosky 1995) of some AML control measures on social welfare. This includes the impact of extra financial transparency on the efficiency of extortionate ‘rent seeking’ by officials and on the suppression of political opposition. Criminological analysis of criminal markets and of different offences—including the impact of evolving technologies in financing crime, crime commission and laundering—can play a part in generating a better baseline for criminal and regulatory policies, and more work needs to be done to link changes in them to AML efforts of various types. Even disregarding the impact that the COVID-19 pandemic will have on resources and priorities around the world, in the final analysis, we need to decide how much investment of resources in both public and private sectors AML efforts merit, and this requires a more critical and detailed analysis than has been common to date. Much expenditure is ‘buried’ in private sector financial and civil liberties costs passed on to customers and shareholders, and in general, it is viewed as merely a regulatory burden rather than as a private or public welfare choice subject to normal cost-benefit criteria. Law enforcement and regulatory expenditures on AML are relatively modest compared with private sector expenditures, and often have been viewed as marginal additions rather than as a substitute for other forms of policing.

In reviewing this area of serious crime governance that lies outside the routine activities of most criminologists, one aim has been to stimulate Asian criminologists and scholars of Asian political economy (as well as those in other parts of the world) to take seriously the task of enhancing their own understanding and their governments’ understandings of criminal economies and of the intended and unintended effects of control efforts on their societies. Potentially, National Risk Assessments and Mutual Evaluation Reports offer an opportunity to think seriously about domestic and transnational crime issues, and their underlying ‘crime scripts’ that include their financing and what happens to the money they make. But if this issue is treated merely as a game theory exercise in how to maximise international AML ratings, this opportunity will be lost. There are no good or easy role models in the Global North or South to copy for this process, and it has both conceptual and empirical challenges, but even if rational people may still fight over what crimes to prioritise, thinking about the financial aspects of crimes offers one route to more evidence-influenced and comprehensive crime control in the interests of a range of publics.

## Data Availability

Not applicable

## Footnotes

1 See further, https://www.longfinance.net/programmes/financial-centre-futures/global-financial-centres-index/gfci-27-explore-data/gfci-27-rank/. These data have begun to take into account the impact of the Hong Kong protests and responses to them upon the perceived independence and stability of Hong Kong. They do not reflect the impact of COVID-19 on both trade and financial investment, and its currently unclear short- and longer term impact on corruption, on the difficulties of moving illicit commodities and of laundering the proceeds.

2 Curiously, there is comparatively little evidence of such overseas funds being used to legitimise major industrial or service investment overseas: it typically appears to be a form of conspicuous consumption and property holdings in housing, cars and planes.

3 https://www.fatf-gafi.org/publications/fatfgeneral/documents/objectives-2019-2020.html

4 The traditional UK mantra of evidence-led policy is less often heard around the world (or even in the UK) at the time of writing, but precautionary policies rest on *predictions* of harm which should use the best evidence from past events: but unlike controlled trials in physical medicine or some policing experiments, the conditions under which they operate are not always replications thereof. This is a broader issue that is outside scope for this article.

5 The US Department of Justice estimates US$4.5 billion was misappropriated from Malaysia’s 1MDB sovereign fund between 2009 and 2014. The US and Malaysian authorities state that over US$1 billion stolen from 1MDB flowed into Najib’s personal banks accounts.

6 For the 2019 EU reports, see https://ec.europa.eu/info/sites/info/files/supranational_risk_assessment_of_the_money_laundering_and_terrorist_financing_risks_affecting_the_union.pdf; https://ec.europa.eu/info/sites/info/files/supranational_risk_assessment_of_the_money_laundering_and_terrorist_financing_risks_affecting_the_union_-_annex.pdf.

7 A view radically different from the now conventional individual or business crime survey which seeks to depersonalise the ‘desired action’ part of the judgement when measuring the ‘true’ levels of crime.

8 If Money Laundering Reporting Officers (MLROs) are *expecting* action on their reports, then apart from numerically relatively low-reporting countries like Switzerland, they should adjust their expectations! Nevertheless, many who report suspicions (and other financial crimes) do want something to be done as a consequence, since otherwise it is not obvious what the value of the system is, except in providing employment for compliance officers at a largely concealed but nevertheless real cost to the public and shareholders.

9 Many other offences—e.g. fraud, rape—in which victims genuinely *feel* harmed may turn out not to be offences, in which the requisite level of criminal intent is judged to exist. But this is beyond the scope of this article.

10 Though https://homeofficemedia.blog.gov.uk/2017/12/11/economic-crime-factsheet/ states: “The scale of money laundering impacting the UK is likely to exceed £90 billion a year – this represents the proceeds of a range of exploitative and violent crimes, including large scale drug dealing and people trafficking. In 2016, 1,435 people were convicted of money laundering in England and Wales. The Government established the Joint Money Laundering Intelligence Taskforce in 2015 to tackle the issue. Between May 2016 and March 2017, they contributed to over 1000 bank-led investigations into suspect customers, the closure of more than 450 suspicious bank accounts, and the freezing of £7 million in suspected criminal funds.”

11 I should declare an interest as a member of the UK Crime Statistics Advisory Committee.

12 One could, for example, predict higher and more critical scrutiny for Lebanese AML efforts in the aftermath of Carlos Ghosn’s flight there from ‘home custody’ in Japan in 2020, as there has already been in the context of Hezbollah organised crime and terror finance activities.

13 Armstrong, M. (2016). *The global extent of the Panama Papers leak*. *Statista*. Statista Inc.. Accessed: December 05, 2019. https://www.statista.com/chart/4606/the-global-extent-of-the-panama-papers-leak/)

14 Buchholz, K. (2019). *The Most Notorious Embezzlers Among State Leaders*. *Statista*. Statista Inc.. Accessed: December 05, 2019. https://www.statista.com/chart/19265/most-notorious-embezzlers-among-state-leaders/

15 See the work of the International Consortium of Investigative Journalists and individual stories, e.g. for a Eurasian story, https://www.bloomberg.com/news/features/2020-03-05/crooked-banker-tapped-professionals-to-manage-web-of-dirty-money.

16 On November 8, 2019, the Financial Crimes Enforcement Network reissued its Geographic Targeting Orders requiring US title insurance companies to identify the natural persons behind legal entities used in purchases of residential real estate performed without a bank loan or similar form of external financing. The monetary threshold remains at $300,000, and the nine districts remain the same. The GTOs cover purchases involving virtual currency as well as “fiat” currency, wires, personal or business checks, cashier’s checks, certified checks, traveller’s checks, a money order in any form or a fund transfer. https://www.fincen.gov/sites/default/files/shared/Real%20Estate%20GTO%20GENERIC_111518_FINAL%20508.pdf. For a discussion, see McPherson (2017).
